# The impact of fibrotic diseases on global mortality from 1990 to 2019

**DOI:** 10.1186/s12967-023-04690-7

**Published:** 2023-11-16

**Authors:** Henricus A. M. Mutsaers, Camilla Merrild, Rikke Nørregaard, Oleguer Plana-Ripoll

**Affiliations:** 1 Department of Clinical Medicine, Aarhus University, Palle Juul-Jensens Boulevard 99, 8200 Aarhus N, Denmark; 2 Department of Renal Medicine, Aarhus University Hospital, Aarhus, Denmark; 3 Department of Clinical Epidemiology, Aarhus University and Aarhus University Hospital, Aarhus, Denmark

To the Editor,

Fibrosis, characterized by the excessive production and accumulation of extracellular matrix (ECM) proteins, is an integral part of numerous chronic diseases affecting vital organs such as the lungs, liver, heart, and kidneys [1]. Despite the diversity in their etiological underpinnings and clinical presentations, these disorders all lead to a common process of tissue remodeling and scarring. This results in the deterioration of organ structure, functional impairment, and ultimately organ failure, often requiring transplantation. While there has been a long-standing notion that fibrotic diseases might account for up to 45% of worldwide deaths, this estimate has lacked solid epidemiological backing. To address this knowledge gap, we turned to the 2019 Global Burden of Disease (GBD) study (https://www.healthdata.org) [2], aiming to uncover the actual impact of fibrotic diseases on global mortality.

From the myriad causes of death documented in the GBD, we specifically focused on conditions connected to ECM remodeling (Fig. 1A). Based on these data, a conservative estimate posits that fibrotic diseases contributed to 16.5% of all global deaths in 1990, and this percentage steadily increased over time to 17.8% in 2019 (Fig. 1A). However, emerging insights indicate that the majority of neoplasms should also be categorized as fibrotic disorders, as fibrosis plays a key role in tumor growth and metastasis [3–5]. When accounting for neoplasms, excluding acute lymphoid leukemia and acute myeloid leukemia, the overall impact of fibrotic diseases on global deaths in 1990 was 28.7%, which subsequently rose to 35.4% in 2019 (Fig. 1A, B). Among all fibrotic disorders, neoplasms and chronic obstructive pulmonary disease consistently ranked as the primary contributors to global mortality during this period (Fig. 1A). In contrast, the impact of various infectious diseases declined over time. For instance, tuberculosis, a significant contributor in 1990, saw its contribution nearly halved by 2019 (Fig. 1A, B), reflecting changing patterns in the global disease landscape over the years.

**Fig. 1:**
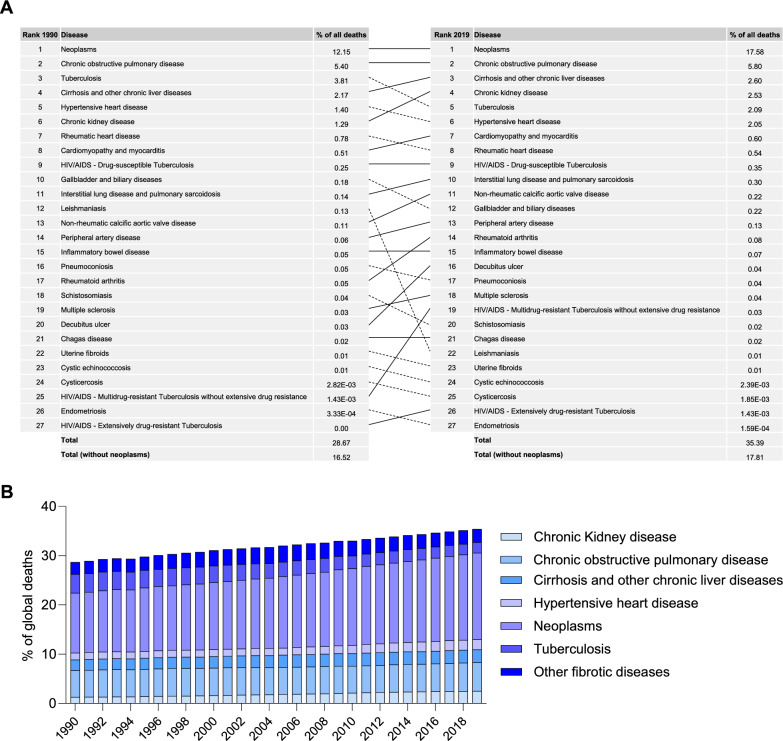
Impact of Fibrotic Diseases on Global Mortality from 1990 to 2019. **A** Selected causes of death and their ranking in 1990 and 2019, based on their percentage contribution to global deaths. **B** Trend of percentage of global deaths attributable to fibrotic diseases from 1990 to 2019

While the impact of fibrotic disorders on global mortality might be smaller than previously estimated, and we recognize that certain deaths involve factors beyond ECM remodeling and fibrosis, it remains evident that fibrotic diseases still contribute significantly to global mortality. This underscores the necessity for sustained research efforts aimed at developing effective antifibrotic treatments, as this critical need remains largely unaddressed.

## Data Availability

All data generated or analysed during this study are included in this published article.

## Abbreviations

ECM: Extracellular matrix
GBD: Global Burden of Disease
